# Genomes of the class *Erysipelotrichia* clarify the firmicute origin of the class *Mollicutes*

**DOI:** 10.1099/ijs.0.048983-0

**Published:** 2013-07

**Authors:** James J. Davis, Fangfang Xia, Ross A. Overbeek, Gary J. Olsen

**Affiliations:** 1 Department of Microbiology and Institute for Genomic Biology, University of Illinois at Urbana-Champaign, USA; 2 Argonne National Laboratory, Argonne IL, USA; 3 Fellowship for Interpretation of Genomes, Burr Ridge, IL, USA; 4 Center for Biophysics and Computational Biology, University of Illinois at Urbana-Champaign, USA

## Abstract

The tree of life is paramount for achieving an integrated understanding of microbial evolution and the relationships between physiology, genealogy and genomics. It provides the framework for interpreting environmental sequence data, whether applied to microbial ecology or to human health. However, there remain many instances where there is ambiguity in our understanding of the phylogeny of major lineages, and/or confounding nomenclature. Here we apply recent genomic sequence data to examine the evolutionary history of members of the classes *Mollicutes* (phylum *Tenericutes*) and *Erysipelotrichia* (phylum *Firmicutes*). Consistent with previous analyses, we find evidence of a specific relationship between them in molecular phylogenies and signatures of the 16S rRNA, 23S rRNA, ribosomal proteins and aminoacyl-tRNA synthetase proteins. Furthermore, by mapping functions over the phylogenetic tree we find that the erysipelotrichia lineages are involved in various stages of genomic reduction, having lost (often repeatedly) a variety of metabolic functions and the ability to form endospores. Although molecular phylogeny has driven numerous taxonomic revisions, we find it puzzling that the most recent taxonomic revision of the phyla *Firmicutes* and *Tenericutes* has further separated them into distinct phyla, rather than reflecting their common roots.

## Introduction

The universal tree of life lies at the heart of our understanding of the evolution of life on Earth, and it serves as a piece of essential infrastructure from which comparisons can be made and hypotheses can be generated. To this end, major efforts have been undertaken to provide reference trees, alignments and tools to guide phylogeny-based scientific inquiry (Alm *et al.*, 2005; Cannone *et al.*, 2002; DeSantis *et al.*, 2006; Felsenstein, 1989; Lane *et al.*, 1985; Letunic & Bork, 2007; Ludwig *et al.*, 2004; Maidak *et al.*, 1994; Overbeek *et al.*, 2005; Schloss *et al.*, 2009; Woese & Fox, 1977). The utility of these tools is perhaps most readily seen in environmental surveys and microbiome analyses wherein sequence data are organized in the context of our prior knowledge of the tree (Lane *et al.*, 1985). In order to ensure the accuracy of such studies, we should seek the best possible tree, and a taxonomy that reflects the tree.

Organisms with rapid evolutionary tempos are often major sources of ambiguity in the tree of life (Simpson, 1944). Among bacteria, this often happens in endosymbionts and parasites that have gone through major population bottlenecks (e.g. Andersson & Kurland, 1998; Moran, 1996; Woese *et al.*, 1985). The rapid accumulation of mutations in the genomes of these lineages results in long branch lengths, which increase random noise and potentially systematic errors, making their ancestral nodes difficult to place with accuracy (Felsenstein, 1978). One of the most noteworthy taxa of this type is the mycoplasma. These bacteria are often parasitic, infecting a wide range of hosts including vertebrates, insects and plants. They have undergone, sometimes drastic, genomic reductions, with some members having only 500 genes. The functions lost include biosynthetic pathways, DNA repair, sporulation and cell wall biosynthesis (e.g. Fraser *et al.*, 1995; Razin, 2006; Ogawa *et al.*, 2011).

In the 1970s, prior to the widespread use of molecular phylogeny methods, all wall-less ‘prokaryotes’ were considered to be related (Darland *et al.*, 1970; Edward & Freundt, 1967; Freundt, 1974; Gibbons & Murray, 1978). However, in the 1980s, Woese and colleagues used the16S rRNA molecule to demonstrate that the absence of the cell wall was a poor phylogenetic marker, showing that the mycoplasma are related to the low G+C Gram-positive (‘firmicute’) bacilli, and that the thermoplasma are members of the *Archaea* (Woese *et al.*, 1980). This observation was later refined when Weisburg *et al.* (1989) discovered a specific relationship between the mycoplasma and an obscure group of low G+C Gram-positives that includes *Clostridium innocuum*, *Clostridium ramosum*, *Erysipelothrix rhusiopathiae* and *Lactobacillus catenaforme*. They dubbed these organisms the ‘walled relatives’ of the mycoplasma (Weisburg *et al.*, 1989).

For many years, the ribosomal phylogeny was used to guide the taxonomy of the mycoplasma and low G+C Gram-positive bacteria. Although all mycoplasma lineages were binned into the single class *Mollicutes* (‘mollis’ for soft or pliable and ‘cutis’ for skin or cell wall) (Edward & Freundt, 1967), they were considered to be members of the phylum *Firmicutes* (e.g. Garrity *et al.*, 2005). More recently, the low G+C Gram-positive members of the phylum *Firmicutes* were divided into three classes: *Bacilli*, *Clostridia* and *Erysipelotrichia* (Ludwig *et al.*, 2009). Members of the class *Erysipelotrichia*, which were named for *Erysipelothrix rhusiopathiae*, are the ‘walled relatives’ of Weisburg *et al.* (1989). However, at the same time, based on the absence of the cell wall and phylogenetic data from non-ribosomal molecules, the mollicutes were designated as belonging to the independent phylum *Tenericutes* (‘tener’ for soft or tender) (Ludwig *et al.*, 2009; *sensu* Murray, 1984). Thus, any relationship between the mollicutes and the firmicutes is obscured by the current nomenclature.

Recently, largely through the efforts of the Human Microbiome Project, many members of the class *Erysipelotrichia* have been isolated and their genomes have been sequenced (Nelson *et al.*, 2010; Turnbaugh *et al.*, 2007). This raises the question: do the new sequence data identify the ‘walled relatives’ of the mycoplasma? If so, what biological properties are common to the related groups? In this study, we examine the evolutionary history of the mollicutes and erysipelotrichia at the molecular level. We search for the appropriate placement of their ancestral node(s), and we explore how the fast-clock evolutionary dynamic of the mollicutes may help us to better understand the evolution and ecology of the erysipelotrichia.

## Methods

### 

#### Alignment generation and curation.

##### Data acquisition.

Sequences for all genes and proteins were downloaded from the SEED database using the tools of the Sapling Server (Aziz *et al.*, 2012; Disz *et al.*, 2010; Overbeek *et al.*, 2005), or from the NCBI ftp server (Wheeler *et al.*, 2007) in March 2012.

##### DNA alignments.

Our 16S rRNA gene alignments were made by first creating an infernal alignment (Nawrocki *et al.*, 2009) using the RDP aligner tool (Cole *et al.*, 2009). The same sequences were also aligned using the mothur package (Schloss *et al.*, 2009) and using the Greengenes template 16S alignment (DeSantis *et al.*, 2006). The mothur and infernal alignments were then merged using the tornado tool (Sipos *et al.*, 2010), which resulted in a modest improvement in the likelihood value for the tree (data not shown). The alignment was then trimmed to the first column having greater than 33 % nucleotide conservation. Large insertions were masked (i.e. omitted from subsequent steps). Variable regions, defined as at least 10 consecutive columns having less than 33 % nucleotide conservation, were extracted and realigned using the mafft einsi tool (Katoh *et al.*, 2002), and reinserted into the alignment. This also results in a very modest improvement in the likelihood score for the tree (data not shown). Insertions that occur in only one of the sequences were masked.

The 23S rRNA gene alignment was created as described above, except that instead of using the RDP, it was seeded with an infernal covariance model (Rivas & Eddy, 2008). Mothur was used as above with a published reference alignment (Cannone *et al.*, 2002).

##### Protein alignments.

Protein alignments representing all of the sequenced organisms in the SEED database were made by first creating alignments from the protein subsystems (Overbeek *et al.*, 2005) using mafft (Katoh *et al.*, 2002). These subsystem-based alignments were used with psi-blast to search all of the sequenced genomes in the SEED database (Altschul *et al.*, 1997). A protein was added to the growing alignment if it matched the profile with an E-value ≥0.01 and a per cent identity ≥15 %. Sequences with major length variations within the conserved portion of the alignment were excluded. Alignments were initially made using mafft and curated with the same realignment of variable regions and masking as described above. When sequences for more than one strain of a species exist, one of the genomes was chosen to represent the species in the protein trees. However, when a specific protein was absent, or of poor quality, in the representative genome, the species sampling was maintained (when possible by substituting the corresponding gene from another strain). The ribosomal protein concatenation was made by first aligning 34 universal ribosomal proteins (Roberts *et al.*, 2008) as described above, and then concatenating these alignments.

For the aminoacyl-tRNA synthetase (AARS) protein trees, the list of organisms was reduced to exclude multiple strains of a species and AARS proteins that had greater than 97 % amino acid identity. All of the sequenced mollicutes and erysipelotrichia, and the available archaea and eukarya, were used. This resulted in most trees having ~1300 taxa. We then computed alignments as above and maximum-likelihood trees (below) for each AARS protein (the α- and β-subunits of Phe-RS alignments were concatenated prior to phylogenetic analysis). Trees shown in Fig. S1 (available in IJSEM Online) include full AARS subtrees, which are necessary and sufficient to cover all of the taxa of the classes *Mollicutes* and *Erysipelotrichia*. In several cases where the classes *Mollicutes* and *Erysipelotrichia* are monophyletic, we provide the 50 closest organisms (based on tree distance) to each taxon as a frame of reference.

#### Tree generation.

Maximum-likelihood trees were made from alignments using RAxML (Stamatakis, 2006) with either the general time reversible model with the gamma distribution model of rate heterogeneity for nucleotide alignments (Tavaré, 1986; Yang, 1996) or the WAG model for amino acid alignments (Whelan & Goldman, 2001). Bootstrap values for small trees with less than 400 taxa were computed using the tools of the Sapling Server (Disz *et al.*, 2010), which invoke a bootstrap that is identical to that of the seqboot program (Felsenstein, 1989). For subtrees greater than or equal to 400 taxa, the RAxML rapid bootstrapping algorithm (Stamatakis *et al.*, 2008) was used to reduce the computation time. Unless otherwise indicated, we performed 100 bootstrap resamplings on all trees. Trees were rendered using the tools of the SEED Sapling server (Disz *et al.*, 2010).

#### AARS tree comparisons.

The AARS protein trees were compared by first rooting the tree on a eukaryaryl or *Aquifex* version of the protein. Then each tree was divided into every possible subtree and the minimum number of subtrees necessary to describe a given phylogenetic group was computed. Since horizontal gene transfer is common in the AARS trees, we allowed up to five taxonomically unrelated sequences to be part of a given group before we considered it to be polyphyletic.

#### Signature analysis.

##### 16S signature analysis.

In order to identify the regions of the 16S rRNA gene that characterize the evolution of the mollicutes and low G+C Gram-positives, we realigned the 16S rRNA genes of all bacterial species in the SEED database against a consensus secondary structure using the program ssu-align, and the frequency of gaps at each position was overlaid onto a reference 16S rRNA gene secondary sequence diagram using the program ssu-draw (Nawrocki, 2009).

##### Kovbasa method.

We analysed primary structure signatures by using the method of Kovbasa (1995). Briefly, this method considers all of the different non-gap characters in an alignment column to be the coordinates of a vector. For a nucleotide alignment, the vector has four components, and for an amino acid alignment the vector has 20 components. Two groups of organisms are selected for comparison and the characters in a given alignment column for group A become the vector components for vector A, and the characters in the alignment column for group B become the components for vector B. The difference between the vectors in the column becomes the measure of signature strength. If two groups differ completely in their nucleotide or amino acid usage, their vectors will be orthogonal. The Kovbasa method then converts these vector differences into a signature value (with 2 being the highest value, indicating that the two groups are completely different).

We made several slight modifications to the Kovbasa method to make it more suitable for studying the mollicutes. First, to prevent columns with a small number of characters from having a strong signature value, we multiplied each signature value by the fraction of total nongap characters found in each column. One characteristic of Kovbasa’s method is that the evolutionary conservation of the characters in a group is not essential for a high signature value. For example, if for a given column group A = [A, A, A, A] and group B = [G, G, G, G], then the column will have a signature value of 2; and likewise, if group A = [A, T, A, T] and group B = [G, C, G, C], the column will also have a value of 2 because the two groups are completely different. Since the columns that are conserved among the mollicutes provide more information about the placement of their ancestral node, we weighted the signature values so that columns with conservation will provide the highest signature values. For a given column, we first computed the Kovbasa value. Then we computed the frequency of each character in each group. We took the largest frequency found in either group, and subtracted the frequency of the same character in the corresponding group from this. That is, if group A = [W, W, W, Y] and group B = [A, L, V, W] then the major character is ‘W’ from group A, which occurs at a frequency of 0.75, and we subtract the frequency of ‘W’ in group B (0.25) from this giving a value of 0.5. This is then divided by 2 and multiplied with the original signature value in order to give a number between 0 (representing no signature strength) and 1 (representing strong signature strength and strong column conservation in at least one group). The behaviour of the Kovbasa signature and our modifications are detailed in Table S1.

##### Signature comparisons of bacterial phyla.

To quantify the relationship between the phylum *Tenericutes* and the other bacterial phyla, we used the NCBI taxonomy (Sayers *et al.*, 2011) to guide the formation of groups for signature analysis. Bacterial phyla with fewer than 20 sequenced genomes were not used in the signature analysis. Random samples of 10 taxa from each of two phyla were used to calculate the modified Kovbasa signature scores described above, and the signature scores were averaged over the columns in the alignment. The average signature scores reported are the mean±sd of 1000 random samplings. A score is reported as being within two standard deviations of the lowest score if it is within two times the square root of the sum of the squared individual standard deviations. Although this measure comes with numerous caveats, if anything it overstates the number of cases in which a phylum other than the *Firmicutes* might be closest to the phylum *Tenericutes*.

#### Genomic analyses.

We searched for genes encoding purine biosynthesis, pyrimidine biosynthesis, fatty acid biosynthesis, arginine biosynthesis, tryptophan biosynthesis and the formation of endospores in the erysipelotrichia genomes. To do this, we obtained representative genes from within the low G+C Gram-positives by searching the KEGG database (Ogata *et al.*, 1999), the SEED subsystems and trees (Overbeek *et al.*, 2005), and the *Bacillus subtilis* genome (Kunst *et al.*, 1997). These genes were used to conduct psi-blast, blastp, and tblastn (Altschul *et al.*, 1997) searches against the erysipelotrichia genomes. When matching sequences were found in members of the class *Erysipelotrichia*, they were used to search against the genomes of organisms lacking a homologue as well.

Codon usage analyses were performed as described by Davis & Olsen (2010, 2011). We compared each erysipelotrichia gene to the modal codon usage of each mollicute genome (Davis & Olsen, 2010). Each comparison is based upon a chi-squared test with *P*≥0.1 being considered to be a match (i.e. the erysipelotrichia gene is not significantly different from the codon usage of the particular mollicute). The comparison of the number of genes in each genome with foreign codon usage was computed as in (Davis & Olsen, 2011). This was done by dividing genomes into native (likely to be vertically inherited) and non-native (likely to be horizontally acquired) subsets.

## Results

### Ribosomal phylogeny

In order to elucidate the evolutionary history of members of the classes *Mollicutes* and *Erysipelotrichia*, we started by examining their phylogeny based upon the components of the ribosome. Many studies have documented the 16S rRNA gene similarities between the mollicutes and low G+C Gram-positive bacteria (Ciccarelli *et al.*, 2006; Collins *et al.*, 1994; Downes *et al.*, 2000; Johansson & Pettersson, 2002; Ogawa *et al.*, 2011; Razin, 2006; Turnbaugh *et al.*, 2008; Weisburg *et al.*, 1989; Woese *et al.*, 1980; Wu & Eisen 2008; Wu *et al.*, 2009). Also, several studies have analysed the 23S rRNA gene and ribosomal proteins (RP) of the mollicutes (Martini *et al.*, 2007; Ogawa *et al.*, 2011; Oshima & Nishida, 2007; Zhao *et al.*, 2005). We build upon these studies by incorporating sequence data from the currently available erysipelotrichia genomes.

**Fig. 1.**
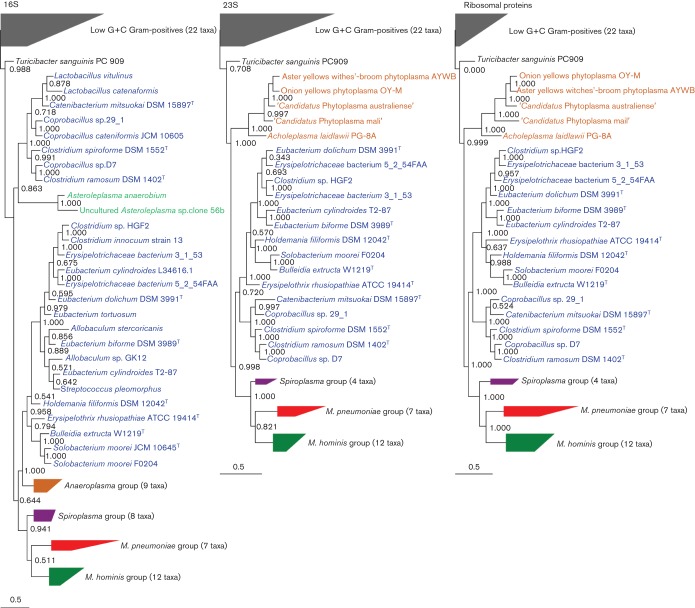
The ribosomal phylogeny of the mollicutes and low G+C Gram-positive bacteria. Previously described groups are coloured (Weisburg *et al.*, 1989). Members of the class *Erysipelotrichia* are shown in blue, the anaeroplasma group is shown in orange, the asteroleplasma group is shown in teal, the spiroplasma group is shown in purple, the *Mycoplasma hominis* group is shown in green and the *Mycoplasma pneumoniae* group is shown in red. Other low G+C Gram-positives are shown in grey. A wedge depicts taxonomic groups that have been collapsed. The top and bottom of the wedge describes the longest and shortest branch lengths found in each group. The total number of taxa is shown in parentheses. The root position for each tree is arbitrary. All trees are maximum-likelihood. Bootstrap values are for 1000 replicates. Bars, 0.5 substitutions per position. Fully expanded trees are shown in Fig. S2.

We examined the genomes of 83 diverse representatives of the class Mollicutes and Low G+C Gram-positive bacteria, including all of the available erysipelotrichia genomes (Table S2). For each genome, we computed alignments and trees for the 16S and 23S rRNA genes. In the case of the 16S alignment, we also included 16S rRNA gene sequences from key organisms for which genomic data are not yet available (Table S2). We also computed alignments for the 34 universal RP (Roberts *et al.*, 2008), and concatenated them (Fig. 1).

Although phylogenetic sampling and phylogenetic inference tools have improved greatly, the 16S rRNA gene tree shows the same four major clusters of mollicutes described over 20 years ago by Weisburg and colleagues (1989) (Fig. 1a): the *Mycoplasma hominis* group; the *Mycoplasma pneumoniae* group, which includes the genus *Ureaplasma*; the spiroplasma group, which includes *Mycoplasma mycoides*, *Mycoplasma capricolum* and the genera *Entomoplasma* and *Mesoplasma*; and the anaeroplasma group, which includes the genera *Acholeplasma* and *Phytoplasma**.* There are also several deep lineages of the class *Mollicutes* that include the uncultivated genera ‘*Candidatus* Bacilloplasma’, ‘*Candidatus* Hepatoplasma’ and ‘*Candidatus* Lumbricincola’ (Fig. S3) (Nechitaylo *et al.*, 2009; Kostanjšek *et al.*, 2007; Wang *et al.*, 2004). The asteroleplasma, which are another group of wall-less mycoplasma-like organisms appear to descend from a node within the class *Erysipelotrichia*. The distinction between *Asteroleplasma anaerobium* and the other members of the class *Mollicutes* was noted previously (e.g. Johansson & Pettersson, 2002; Stephens *et al.*, 1985; Weisburg *et al.*, 1989); however, very little is known about the genus *Asteroleplasma*, and a genome sequence is not yet available to better resolve its relationships. The 23S rRNA gene and RP trees are nearly identical (Fig. 1b, c) and they resemble the 16S rRNA gene tree. In the 23S rRNA gene and RP trees, the erysipelotrichia split the mollicutes, whereas in the 16S rRNA gene tree, the erysipelotrichia share a node with the anaeroplasma group. The location of the anaeroplasma node has strong bootstrap support in all three trees, and masking variable columns in the alignments and changing models of nucleotide substitution did not lead to a reconciliation in these tree topologies. A tree based upon the concatenation of the 16S rRNA gene and 23S rRNA gene alignments has topology resembling the 23S rRNA gene tree (data not shown).

Several aspects of the current taxonomy deserve note. First, the genera found wholly within the class *Erysipelotrichia* tend to be phylogenetically consistent. Second, the genus *Turicibacter* is classified as a member of the class *Erysipelotrichia*, and while it is indeed similar to the erysipelotrichia, it occupies a deeper branch that is outside of the main erysipelotrichia group; that is, it is best viewed as being peripherally related (Ogawa *et al.*, 2011). Third, there remain many species that are misclassified as clostridia, eubacteria, streptococci and lactobacilli interspersed within the erysipelotrichia. Finally, all of the trees in Fig. 1 indicate that the mollicutes and erysipelotrichia are related and that they share a common evolutionary root within the low G+C Gram-positive bacteria. These relationships are also supported by larger 16S and 23S rRNA gene trees covering all of the complete genomes in the SEED database (data not shown).

### Signature analysis of the 16S rRNA molecule

**Fig. 2.**
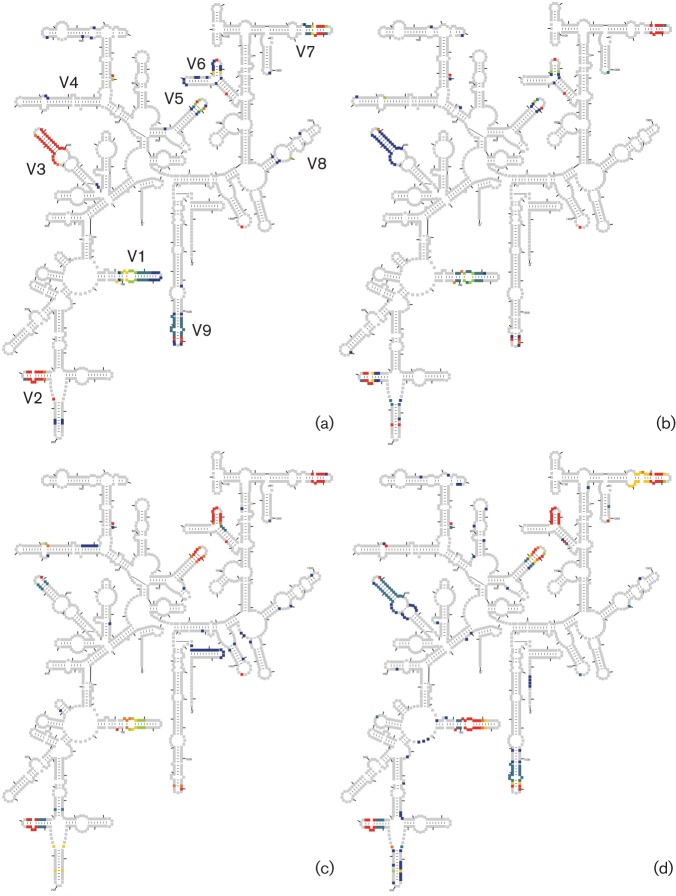
The frequency of gaps occurring at positions in the 16S rRNA gene sequence. 16S alignments were made for a) the class *Clostridia* (82 individual species), b) the class *Bacilli* (76 individual species), c) the class *Erysipelotrichia* (26 organisms with more than one strain of a species) and d) the phylum *Tenericutes* (56 organisms with more than one strain of a species). Each block in the diagram represents a nucleotide position and colouring is as follows: grey, a gap occurring at a frequency of less than 0.001; violet, a gap occurring at a frequency of less than 0.05; blue, a gap occurring a frequency of less than 0.2; green, a gap occurring at a frequency of less than 0.35; yellow, a gap occurring at a frequency of less than 0.5; orange, a gap occurring at a frequency of less than 0.75; and red, a gap occurring at a frequency of greater than 0.75. Variable regions V1–V9 are indicated for reference (Böttger, 1996).

The classic molecular phylogeny literature often paired the use of trees and signature analysis in order to make evolutionary inferences (e.g. Woese, 1987). Signature analysis is performed by searching for sequence characteristics that link some organisms, while distinguishing them from others. The factor that distinguishes signatures from more general phylogenetic analyses is the focus on the most slowly changing characteristics. The 16S rRNA molecules from Table S2 were aligned based on secondary structure, and the presence or absence of a nucleotide at each position of a bacterial consensus 16S rRNA molecule is shown in Fig. 2.

There are regions of the 16S rRNA molecule where gaps are a characteristic signature of members of the classes *Clostridia*, *Bacilli*, *Erysipelotrichia* and *Mollicutes*. These include V1 (*Esherichia coli* positions 61–106), V2 (*Esherichia coli* positions 200–217), V5 (*Esherichia coli* positions 836–850) and V7 (*Esherichia coli* positions 1130–1143), with the gaps in V1 and V5 being more pervasive in the erysipelotrichia and mollicutes. There is also a large gap in V3 (*Esherichia coli* positions 451–480) that is specific to members of the class *Clostridia* and is rare in the other three groups. In the mollicutes and erysipelotrichia, the V6 stem–loop (*Esherichia coli* positions 1025–1036) is completely absent in all of the organisms except *Lactobacillus catenaforme* (*Erysipelotrichia*). This V6 gap includes *Clostridium sp.* HGF2 and the genus *Asteroleplasma*, but excludes the genus *Turicibacter*. The V6 gap linking the classes *Mollicutes* and *Erysipelotrichia* is quite rare. When we searched the 16S rRNA gene sequences of 628 individual bacterial species in the SEED database (Overbeek *et al.*, 2005) we found only 6 other instances where other organisms were missing the V6 stem–loop (*Calditerrivibrio nitroreducens*, *Deferribacter desulfuricans*, *Desulfobulbus propionicus*, *Dictyoglomus thermophilum*, *Thermosipho africanus* and *Slackia heliotrinireducens*). Thus, the absence of the V6 stem–loop that pervades the mollicutes and erysipelotrichia appears to be a signature of their shared evolutionary origin.

### AARS phylogeny

The evolutionary patterns of the AARS proteins are well characterized and generally follow the ribosomal phylogeny, albeit with significant instances of horizontal gene transfer and gene duplication (Doolittle & Handy, 1998; Woese *et al.*, 2000). In a previous study, the AARS protein trees were used as supporting evidence for the establishment of the phylum *Tenericutes* because the class *Mollicutes* appeared to be an entirely exclusive group in many of these trees (Ludwig & Schleifer, 2005; Ludwig *et al.*, 2009). However, no genomes belonging to members of the class Erysipelotrichia were available at that time.

To obtain a more detailed understanding of the relationship between the classes *Mollicutes* and *Erysipelotrichia*, we performed a phylogenetic analysis of the 20 AARS proteins found in the members of the class *Mollicutes*. For each AARS protein, we made alignments and trees containing all of the species with sequenced genomes in the SEED database. Then, for each tree, we ask whether the mollicutes and erysipelotrichia group together – a result that would not be expected for members of independent phyla.

Overall, every AARS protein tree except Ser-RS shows evidence of a relationship between the mollicutes and low G+C Gram-positives (Fig. S1). In 8 of the 20 AARS trees, members of the classes *Mollicutes* and *Erysipelotrichia* form monophyletic groups that closely resemble the ribosomal phylogeny: Ala-RS, Asn-RS, Cys-RS, His-RS, Ile-RS, Lys-RS, Phe-RS and Val-RS (we allow minor instances of horizontal gene transfer into the group, but do not allow separation of the members of the group; see Methods). Only the anaeroplasma have a copy of the Gln-RS protein. In this tree, the anaeroplasma are monophyletic and group with the genus *Turicibacter* and other low G+C Gram-positives. In the case of Gly-RS, the mollicute and erysipelotrichia subtree is not monophyletic because it also contains other members of the low G+C Gram-positives. In many of the other AARS trees, horizontal gene transfer has impacted the evolutionary pattern. For instance, in the cases of Glu-RS, Pro-RS, Thr-RS and Trp-RS the members of the class *Erysipelotrichia* split, with one subgroup having the mollicute-like version of the protein and the other subgroup having a different, usually low G+C Gram-positive-like version of the protein. In these cases, the data appear to indicate that a non-mollicute-like version of a synthetase was acquired by the members of the class *Erysipelotrichia*, and then one of the two copies was subsequently lost. In the cases of Asp-RS, Leu-RS, Met-RS and Tyr-RS, the members of the class *Mollicutes* split, and one of these subsets groups with some or all of the members of the class *Erysipelotrichia*. Here the likely evolutionary scenarios are either the acquisition of a new copy of the synthetase by the members of the class *Mollicutes* via horizontal gene transfer, or the accelerated accumulation of mutations in the mollicute lineage that results in an artefactual branching. Finally, in the case of Arg-RS, the members of the class *Mollicutes* do not group with the members of the class *Erysipelotrichia*. Instead, the members of the class *Erysipelotrichia* are monophyletic and group with a subset of the class *Bacilli*, the anaeroplasma share a branch with a different subset of the class *Bacilli* elsewhere in the tree, and the other members of the class *Mollicutes* exist as a separate group.

Although many of the AARS protein trees resemble the ribosomal phylogeny, it is difficult to evaluate which AARS trees provide a more reliable phylogenetic signal since there have been numerous horizontal gene transfer and duplication events that have happened independently in various clades. To do this, we asked whether the AARS trees with a monophyletic mollicute/erysipelotrichia clade are in some objective sense better than the AARS trees that split the proposed clade. To assess the overall reliability of each AARS tree, we evaluated the coherence, or lack of coherence, of seven other well-defined and accepted phylogenetic groups. For example, the members of the class *Actinobacteria* are distributed into three groups in the Ala-RS tree, and into six groups in the Arg-RS tree (Table 1). Our overall assessment of the AARS tree was the sum of the number of subtrees required to encompass the seven phylogenetic groups. The mean number of subtrees per amino acid for the eight AARS trees with a monophyletic Mollicute/Erysipelotrichia group is 19.9±2.6 (sem), while the mean number of subtrees necessary to accommodate the same groups in the AARS trees with a polyphyletic mollicute/erysipelotrichia group is 31.4±2.6, which is significantly larger, though not overwhelmingly so (*P*<0.05). Thus, although the numerous gene transfers in AARS evolution could be used to dismiss any particular tree, the trees supporting the mollicute/erysipelotrichia clade display evidence of an overall lower transfer rate, and therefore can objectively be put forward as the most likely to reflect organismal relationships.

**Table 1.** The number of monophyletic subtrees necessary to describe the mollicute-erysipelotrichia relationship, and a comparison to several other bacterial groups* Taxa: 1, classes *Mollicutes* and *Erysipelotrichia*; 2, phylum *Actinobacteria*; 3, phylum *Bacteroidetes*; 4, phylum *Cyanobacteria*; 5, class *Alphaproteobacteria*; 6, classes *Betaproteobacteria* and *Gammaproteobacteria*; 7, classes *Deltaproteobacteria* and *Epsilonproteobacteria*; 8, phylum *Spirochaetes*. Data for aminoacyl-tRNA synthetases that are underrepresented in certain taxa are not shown.

AARS tree	Taxa								Sum	
	1	2	3	4	5	6	7	8	Monophyletic sets	Polyphyletic sets
Ala	1	3	1	1	1	1	5	2	14	
Arg	3	6	2	2	2	5	12	3		32
Asn	1	1	2	1	–	1	7	2	14	
Asp	6	8	3	1	1	2	5	3		23
Cys	1	2	2	1	6	5	7	4	27	
Glu	2	5	1	2	3	3	10	2		26
His	1	10	2	3	3	3	10	3	34	
Ile	1	3	1	1	3	2	9	2	21	
Leu	6	5	3	1	4	1	11	3		28
Lys†	1	3	1	1	–	1	6	1	13	
Met	5	7	1	2	10	3	8	3		34
Phe‡	1	2	1	1	1	1	6	3	15	
Pro	2	8	2	1	2	2	9	3		27
Ser	6	7	3	1	1	1	11	3		27
Thr	4	7	1	4	3	10	8	3		36
Trp	3	6	8	1	2	12	14	9		52
Tyr	3	5	2	1	2	3	12	4		29
Val	1	5	2	1	2	1	8	2	21	
Mean±se									19.9±2.6	31.4±2.6

* Based on NCBI Taxonomy (Sayers *et al.*, 2009).

† Class-II version.

‡ Concatenation of α and β subunits.

### Signature analysis of the AARS proteins

One reason why there is confusion regarding the phylogeny of the class *Mollicutes* is that members of this class have experienced a large number of changes in their conserved sequences. This increases tree branch lengths and potentially distorts the tree topology (Felsenstein, 1978). This is seen both in the distances between the class *Mollicutes* and other phyla, and in the distances between individual species within the class *Mollicutes*. This is also seen when sequences are compared directly. For example, a bacterial phylum is often defined as a group of organisms that differ by <20 % in their 16S rRNA gene sequences (Hugenholtz *et al.*, 1998); however, it is commonplace for individual mollicute species to differ by this much, even when masking variable columns (e.g. Lane, 1991) (data not shown). Thus, in order to glean information about the ancestry of the class *Mollicutes* it is useful to examine the alignment positions where conservation has been maintained throughout the group. If the members of the class *Mollicutes* have originated from within the low G+C Gram-positives, then the columns that are still conserved within the class *Mollicutes* should most commonly match those of the low G+C Gram-positives. Likewise, there should be few examples where conservation is maintained within the class *Mollicutes*, but is lost or represented by a different character in the low G+C Gram-positives.

To compare these conserved alignment columns, we performed a modified version of the Kovbasa signature analysis method (Kovbasa, 1995; Methods). Briefly, given an alignment and two sets of organisms, the analysis is performed by computing a signature score for each alignment column. The score indicates the degree to which the column distinguishes the two sets of organisms. In particular, a maximal signature is a column in which the values occurring for organisms in one set never occur for organisms of the other set. The scores using the modified Kovbasa function range from 0 being the weakest signature strength (equal usage of characters in the two sets), to 1 being the strongest signature strength (completely distinct characters in the two sets, with full conservation in at least one of the sets).

The analysis was performed using 19 AARS alignments, and 7 phyla (Table 2). For each alignment and phylum we computed a measure of how well the alignment columns act as signatures, separating that phylum from the phylum *Tenericutes*, and these scores were averaged across all columns. This process was carried out for 1000 replicates in which 10 genomes were randomly selected from the phylum and 10 genomes were randomly selected from the phylum *Tenericutes* (in all cases, species were represented by a single strain). For each column in the alignment we computed the signature score reflecting how well the column distinguished the two groups of organisms. The values in Table 2 are the mean±sd for the 1000 replicates. Thus, a low value in the table indicates similarity to the phylum *Tenericutes*.

**Table 2.** Signature analysis of the relationship between the phylum *Tenericutes* and other bacterial phyla for the AARS proteins* na, Not available.

	Bacterial phyla with genome sequences for more than 20 species‡						
Alignment†	*Actinobacteria*	*Bacteroidetes*–*Chlorobi*	*Chlamydiae*-*Verrucomicrobia*	*Cyanobacteria*	*Firmicutes*	*Proteobacteria*	*Spirochaetes*
16S	0.075±0.005	0.071±0.006	0.063±0.004	0.079±0.004	***0.040±0.005***	0.058±0.005	0.056±0.004
Ala-RS	0.110±0.009	0.099±0.007	0.089±0.007	0.126±0.007	***0.067±0.008***	0.095±0.008	**0.082±0.008**
Arg-RS	0.051±0.006	0.086±0.012	0.076±0.008	0.099±0.010	***0.038±0.008***	***0.038±0.005***	0.063±0.011
Asn-RS	0.120±0.009	0.093±0.011	0.095±0.008	0.128±0.008	***0.067±0.015***	**0.091±0.013**	**0.075±0.009**
Asp-RS	0.078±0.013	0.080±0.009	**0.055±0.005**	0.110±0.006	***0.049±0.006***	0.065±0.007	**0.050±0.006**
Cys-RS	0.075±0.006	0.076±0.006	0.067±0.005	0.076±0.004	***0.050±0.005***	**0.054±0.005**	**0.062±0.006**
Glu-RS	0.107±0.013	0.098±0.009	0.081±0.007	0.109±0.008	***0.048±0.008***	0.081±0.008	0.091±0.010
Gly-RS§	0.100±0.007	0.108±0.009	0.072±0.004	na	***0.057±0.007***	na	0.080±0.007
His-RS	0.067±0.009	0.102±0.013	0.055±0.006	0.067±0.007	***0.042±0.005***	**0.053±0.007**	0.082±0.009
Ile-RS	0.086±0.010	0.098±0.005	**0.049±0.007**	0.067±0.004	***0.035±0.007***	0.046±0.005	0.071±0.011
Leu-RS	0.045±0.005	0.041±0.005	**0.029±0.003**	0.059±0.004	***0.024±0.003***	0.042±0.006	0.032±0.003
Lys-II-RS§	0.119±0.015	0.108±0.007	0.076±0.006	0.101±0.009	***0.050±0.007***	0.081±0.007	0.129±0.004
Met-RS	**0.064±0.012**	0.107±0.006	**0.053±0.007**	0.067±0.006	***0.042±0.005***	**0.055±0.012**	0.087±0.010
Phe-RS||	0.104±0.009	0.088±0.008	***0.062±0.006***	0.097±0.008	**0.063±0.007**	**0.069±0.007**	0.092±0.013
Pro-RS	0.149±0.020	**0.081±0.007**	***0.072±0.010***	0.184±0.007	**0.083±0.027**	0.138±0.017	**0.080±0.015**
Ser-RS	0.139±0.025	0.125±0.015	**0.083±0.011**	0.112±0.009	***0.068±0.013***	**0.074±0.010**	0.134±0.022
Thr-RS	0.125±0.014	0.111±0.009	0.087±0.008	0.117±0.010	***0.064±0.010***	0.101±0.010	0.092±0.007
Trp-RS	**0.060±0.006**	0.075±0.008	0.079±0.008	0.098±0.005	***0.049±0.012***	**0.057±0.008**	0.078±0.010
Tyr-RS	0.106±0.013	0.100±0.014	**0.074±0.010**	0.201±0.008	***0.070±0.013***	**0.102±0.018**	0.101±0.014
Val-RS	0.060±0.009	0.063±0.005	**0.046±0.004**	0.072±0.004	***0.039±0.004***	0.050±0.005	0.051±0.004

* Data are the mean±sd (1000 replicates) signature score of the alignment for 10 randomly chosen organisms from each phylum. The closest matching group (fewest mean signature differences per column) in each row is shown in bold italics, and those within two standard deviations are shown in bold.

† Gln-RS is excluded because it is underrepresented in the phylum *Tenericutes*.

‡ Based on NCBI Taxonomy (Sayers *et al.*, 2009).

§ Data may not be representative for all phyla.

||Concatenation of α and β subunits.

For each alignment, the table highlights the taxon with the lowest signature score to the phylum *Tenericutes* (greatest support for closely related, in bold italics), and all taxa with signature scores within two standard deviations of the lowest (plausible candidates for most closely related, in bold). The mean signature values in 18 of the 20 alignments show the phylum *Firmicutes* having a smaller separation from the phylum *Tenericutes* than any of the other six phyla. For the other two alignments, the phylum *Firmicutes* cannot be excluded from being most similar. In summary, all of the AARS alignments are consistent with the members of the phylum *Firmicutes* being the closest relatives to those of the phylum *Tenericutes* and none of the other phyla offer consistent support of an alternative relationship. These results are consistent with the ribosomal phylogeny shown above and with previous studies that have examined this relationship (e.g. Ciccarelli *et al.*, 2006; Collins *et al.*, 1994; Downes *et al.*, 2000; Johansson & Pettersson, 2002; Martini *et al.*, 2007; Ogawa *et al.*, 2011; Razin, 2006; Oshima & Nishida, 2007; Turnbaugh *et al.*, 2008; Weisburg *et al.*, 1989; Woese *et al.*, 1980; Wu & Eisen, 2008; Wu *et al.*, 2009; Zhao *et al.*, 2005).

### Evolutionary characteristics of the class *Erysipelotrichia*

**Fig. 3.**
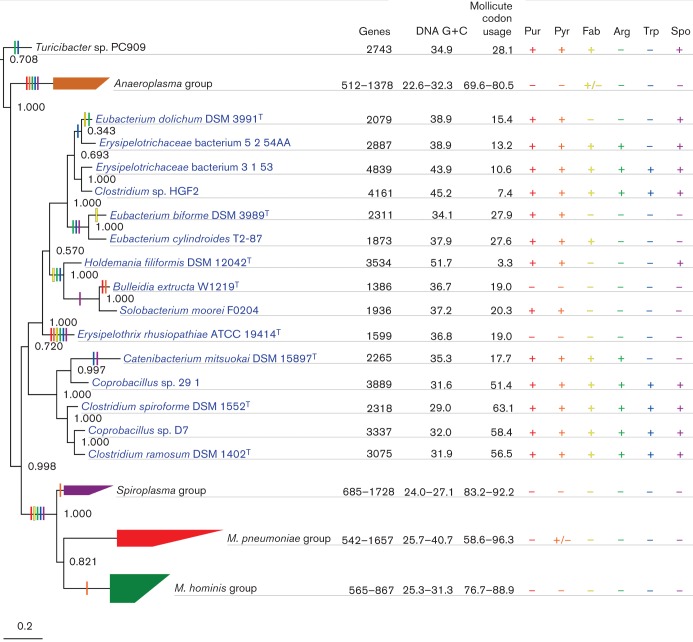
Characteristics of the genomes of members of the classes *Erysipelotrichia* and *Mollicutes*. The 23S rRNA gene tree from Fig. 1(b) is shown. For each genome, the first three columns of data show the number of protein-encoding genes, the mean DNA G+C content for all protein-encoding genes, and the percentage of genes in the genome that match the modal codon usage of any mollicute genome. In each case, collapsed taxa are depicted as a range. The remaining columns indicate whether the given genome has known genes for: purine biosynthesis (Pur, red), pyrimidine biosynthesis (Pyr, orange), fatty acid biosynthesis (Fab, yellow), arginine biosynthesis (Arg, green), tryptophan biosynthesis (Trp, blue) and the formation of endospores (Spo, purple). Presumed pathway losses are indicated by correspondingly coloured vertical bars on the branches in the tree.

If the members of the class *Mollicutes* are indeed specifically related to those of the class *Erysipelotrichia*, then they would be expected to share other evolutionary characteristics beyond those surveyed by gene sequences per se. Since one of the hallmarks of mollicute evolution is the propensity to lose genes, we explored this phenomenon in the genomes of the class *Erysipelotrichia*, where genome reduction has been reported for some of the individual genomes (Ogawa *et al.*, 2011; Turnbaugh *et al.*, 2008). Here we compare the number of protein-encoding genes, DNA G+C contents, codon usages and the presence or absence of the genes for several biosynthetic pathways as a form of illustration (Fig. 3).

The number of protein encoding genes ranges from 1386 in the genome of *Bulleidia extructa* (which is nearly as small as the genome of *Acholeplasma laidlawii*) to 4839 in the genome of *Erysipelotrichaceae* bacterium 3_1_53. Gene content does not follow the phylogeny per se, indicating multiple independent genome reductions. For instance, the genomes of the close relatives *Coprobacillus sp.* D7 and *Clostridium spiroforme* differ by 1019 genes. While it is common to see genomic size differences of this magnitude among closely related bacterial strains (e.g. Perna *et al.*, 2001), much of the observed variation is contributed by genes of atypical (non-native) codon usage. However, even when genes with atypical codon usages are removed from the analysis (see Methods), these *Erysipelotrichaceae* genomes still vary in size by 914 genes. That is, the disparity appears to be predominantly due to gene loss, rather than recent horizontal gene transfer into the larger genome.

Overall, there is considerable variability in the mean DNA G+C contents of protein-encoding genes in the *Erysipelotrichia* genomes. They range from 51.7 % in *Holdemania filiformis* to 29.0 % in *Clostridium spiroforme*, which is lower than that of the wall-less species *Acholeplasma laidlawii* (32.3 %). Having a low DNA G+C content does not always appear to be in step with gene loss. For instance *Coprobacillus sp.* 29_1 has one of the largest genomes (3889 genes) and one of the lowest DNA G+C contents (31.6 %). Overall, the DNA G+C content of the *Erysipelotrichia* genomes is changing rapidly in the tree.

Codon usage is constrained by DNA G+C content but can provide more information about the history of individual genes. In order to see how similar each *Erysipelotrichia* genome is to those of members of the class *Mollicutes*, we first computed the modal codon usages for each of the mollicute genomes. Then we computed the percentage of genes in each *Erysipelotrichia* genome that match any of these mollicute genome modes (Davis & Olsen, 2010) (Fig. 3). There is considerable variability in the percentage of genes in each *Erysipelotrichia* genome matching those in members of the class *Mollicutes*, ranging from 3.3 % of the *Holdemania filiformis* genes to 63.1 % of the *Clostridium spiroforme* genes. So far we have not recognized any property that is more predictive of the more mollicute-like codon usage. Although a low DNA G+C content is necessary for codon usage similarity, it clearly is not sufficient. Likewise, genome size does not appear to be predictive either.

To assess the impact of gene loss in the *Erysipelotrichia* genomes, we searched for genes encoding the proteins for purine biosynthesis, pyrimidine biosynthesis, fatty acid biosynthesis, arginine biosynthesis, tryptophan biosynthesis and the formation of endospores. We chose these processes because they require many genes, so it is more plausible that their presence in the erysipelotrichia is due to vertical inheritance rather than horizontal gene transfer. Furthermore, trees of these proteins tend to be consistent with vertical inheritance (data not shown). When the presence/absence data for these processes are mapped on the tree, it provides information about the extent to which gene loss is occurring independently in each strain. Six of the *Erysipelotrichia* genomes – *Clostridium ramosum*, *Clostridium spiroforme*, *Coprobacillus sp.* D7, *Coprobacillus sp.* 29_1, *Clostridium sp.* HGF2 and *Erysipelotrichaceae* bacterium 3_1_53 – contain the genes for all six processes examined (Fig. 3; Table S3). In the two erysipelotrichia with the smallest genomes, the genes for all six pathways are absent. In the case of the other erysipelotrichia, the presence/absence of the genes for these pathways is indicative of a complex pattern of genome reduction. For instance, fatty acid biosynthesis appears to have been lost independently in the *Erysipelothrix rhusiopathiae*, *Holdemania filiformis*–*Bulleidia extructa*, *Eubacterium biforme* and *Eubacterium dolichum* lineages (presumptive loss events are indicated by coloured vertical bars in Fig. 3). The number of loss events for these functions ranges from 3–7, which is remarkable given that only 17 taxa were available. Overall, multiple independent pathway losses appear to be a hallmark of *Erysipelotrichia* evolution.

Although there are fewer extant genes to compare among the members of the class *Mollicutes*, we observed two instances where gene loss did not follow the tree. *Acholeplasma laidlawii* has retained the genes for fatty acid biosynthesis despite the absence of these genes in the other members of the class *Mollicutes*. Also, *Mycoplasma penetrans* HF-2 has genes for pyrimidine biosynthesis even though it does not have the genes for purine biosynthesis and none of the other members of the class *Mollicutes* appear to have retained these genes.

Several of the erysipelotrichia have been shown to form endospores (Holdeman *et al.*, 1971; Kaneuchi *et al.*, 1979; E. Allen-Vercoe, personal communication; Smith & King, 1962). Although these genomes carry many of the genes previously characterized as being essential for sporulation, such as the genes for dipicolinate synthase (*spoVF* operon) (Traag *et al.*, 2010), we were unable to find genes that are homologous to those of the *Bacillus subtilis* *spoIIIA* operon in any of these genomes. In conjunction with SpoIIQ (which the erysipelotrichia appear to have), the proteins of the *spoIIIA* operon are thought to form a secretion apparatus, or ‘feeding tube’, between the mother cell and the forespore (Camp & Losick, 2009; Doan *et al.*, 2009). Our inability to find these genes indicates that the *spoIIIA* operon is either non-essential, or that there are alternative means by which the *spoIIIA* functions are achieved. To our knowledge, *Eubacterium dolichum* is the endospore-forming organism with the smallest genome (2079 genes), so it may provide a useful model for understanding endospore development.

## Discussion

The reductive evolution of endosymbionts and parasites is a topic of considerable interest within the biological community (e.g. Andersson & Kurland, 1998; Andersson *et al.*, 1998; Moran, 1996; Sagan, 1967; Woese *et al.*, 1985). The class *Mollicutes* represents a particularly acute instance of this phenomenon with many examples of diverse species and hosts, and with interspecies divergences being greater than that of other well-studied host-associated organisms such as members of the genera *Buchnera* and *Rickettsia*. This mode of evolution commonly manifests as exceptionally long branch lengths between species, and is attributable to a very rapid tempo of evolution and/or more ancient divergences between species. Members of the genera *Buchnera* and *Rickettsia* are thought to have become host associated ~250 million years ago and ~180–425 million years ago, respectively (Khachane *et al.*, 2007; Moran *et al.*, 1993; Ochman *et al.*, 1999); whereas the members of the class *Mollicutes* are thought to have diverged from the low G+C Gram-positive bacteria ~600 million years ago, with the anaeroplasma group diverging from the other mollicute groups ~490 million years ago, near the time of the Cambrian explosion (Maniloff, 1996, 2002). Given that the divergences within the class *Mollicutes* are, within measurement error, as deep as their separation(s) from members of the class *Erysipelotrichia*, this suggests that ~490 million years ago would also be an approximate date for the radiation of the class *Erysipelotrichia*. Regardless of the date, these ancient radiations may have resulted from the ability of their ancestors to live in a large diversity of host environments, followed by a piecemeal descent of some lineages into more specialized parasitic associations. This apparently has not yet happened in some of the free-living members of the class *Erysipelotrichia*.

We were not able to resolve the ordering of early events in the splitting of the classes *Mollicutes* and *Erysipelotrichia*. In the 16S rRNA gene tree, the anaeroplasma share a branch with members of the class *Erysipelotrichia*, and in the 23S rRNA gene and RP trees, members of the class *Erysipelotrichia* separate the anaeroplasma from the other mollicute groups (Fig. 1). Both topologies are well supported by bootstrap values, but compositional shifts and the large divergences of some sequences introduce systematic biases that can exceed the variance due to sampling (which is estimated by the bootstrap). These topologies are not reconciled when the most variable columns are masked from the alignment, or when different evolutionary models and treeing algorithms are used (data not shown). Resolving the appropriate location of the anaeroplasma and erysipelotrichia groups would be of interest because the RP and 23S rRNA gene trees indicate that the mollicutes are polyphyletic, which would imply that the cell wall has been lost at least twice within the class *Mollicutes*. This evolutionary scenario is not outlandish; for instance it is supported by the ancillary observation that in the 16S rRNA gene tree, the genus *Asteroleplasma* branch appears to be descending from within the class *Erysipelotrichia* (Fig. 1a), and, furthermore, the cell wall has been lost on separate occasions elsewhere in the tree of life (Darland *et al.*, 1970; Klieneberger, 1934; Lin & Rikihisa, 2003; McCoy & Maurelli, 2006). The genome sequence of *Asteroleplasma anaerobium* would greatly improve our understanding of its phylogenetic placement, and how the cell wall and other features have been lost in the mycoplasma-like organisms.

There has been a trend in the evolutionary literature toward the use of trees that are generated from concatenated protein alignments (Ciccarelli *et al.*, 2006; Wu & Eisen, 2008; Wu *et al.*, 2009). These trees generally provide a reliable average tree topology for the proteins that are chosen. However, this approach is based upon the assumption that the genes for each of the proteins has the same evolutionary history (presumably due to vertical inheritance), or that departures from this assumption are insignificant. It is also subject to bias resulting from the absence of proteins in some lineages, and choosing among paralogous genes. Recent studies using this approach have all shown a close relationship between the class *Mollicutes* and the phylum *Firmicutes* (Ciccarelli *et al.*, 2006; Wu & Eisen, 2008; Wu *et al.*, 2009), and more recently a close relationship between the classes *Mollicutes* and *Erysipelotrichia* (Ogawa *et al.*, 2011). Our data are consistent with these studies, but also highlight the need for analysing individual molecules so that possible contradictory data are not overlooked and the confidence in the tree topology is not overstated. In this way, the ancient details of the *Erysipelotrichia*–*Mollicutes* radiation event may be ascertained as more data become available.

One of the major hallmarks of mollicute evolution is extreme gene loss, which has impacted all of the members of the class *Mollicutes* to varying degrees (e.g. Fraser *et al.*, 1995; Razin, 2006). Genome reduction has also been documented in the class *Erysipelotrichia* (e.g. Chen *et al.*, 2012; Ogawa *et al.*, 2011; Turnbaugh *et al.*, 2008), but unlike the class *Mollicutes*, gene loss appears to have impacted the class *Erysipelotrichia* less uniformly, with some species having rather large genomes and others having quite reduced genomes. Our juxtaposition of genomic data with the phylogenetic tree suggests that much like members of the class *Mollicutes*, the members of the class *Erysipelotrichia* have lost metabolic functions in numerous separate events, with some losses occurring independently in multiple branches of the tree (Fig. 3). Similarly, the DNA G+C contents and codon usages of the protein encoding genes in the *Erysipelotrichia* genomes also vary idiosyncratically. The evolution of these genomic features provides a portrait of the speciation and host adaptation that has occurred in the class *Erysipelotrichia*. This is particularly intriguing in that several members of this group are pathogens or opportunistic pathogens, and many others are commonly found among the human-associated microbiota (e.g. Cornell & Glover, 1925; Downes *et al.*, 2000; Nelson *et al.*, 2010; Turnbaugh *et al.*, 2007).

Although we are unable to resolve the most ancient details, all of our results support the work of Woese and colleagues (Rogers *et al.*, 1985; Weisburg *et al.*, 1989; Woese *et al.*, 1980; Woese *et al.*, 1985) and other groups (e.g. Ciccarelli *et al.*, 2006; Collins *et al.*, 1994; Johansson & Pettersson, 2002; Ogawa *et al.*, 2011; Razin, 2006; Turnbaugh *et al.*, 2008; Wu & Eisen, 2008; Wu *et al.*, 2009) that indicated that the members of the class *Mollicutes* are phylogenetically embedded within the low G+C Gram-positive bacteria, in general, and are related to the members of the class *Erysipelotrichia* in particular. Despite this evolutionary relationship, the class *Mollicutes* and the phylum *Firmicutes* have been actively separated taxonomically (Ludwig & Schleifer, 2005; Ludwig *et al.*, 2009). While taxonomy, by definition, is not constrained to reflect phylogeny, we have seen a tremendous tendency toward their unification in the past 40 years as molecular analyses have revealed relationships that were thought by some to be unknowable. Thus it is strange to see this particular relationship increasingly obfuscated. The data presented in this study are not consistent with the class *Mollicutes* being a separate bacterial phylogenetic group representing an independent divergence from an ancestral bacterial lineage. As expected for correct hypotheses, the support for the relationship between the class *Mollicutes* and low G+C Gram-positives has increased with additional data, in this case the *Erysipelotrichia* genome sequences.
